# Exploring the Value of Hosting a Grassroots Neuroscience Workshop That Facilitates Near-Peer Engagement Between Medical Students and High School Students (Local Brain Bee Participants) in a Developing Country in the Caribbean

**DOI:** 10.7759/cureus.36222

**Published:** 2023-03-16

**Authors:** Gabrielle Walcott-Bedeau, Ernesto Navarro Garcia, Hiba Al-Rubaye, Ariana Stergiou, Kesava Mandalaneni

**Affiliations:** 1 Department of Neuroscience, St. George's University School of Medicine, St. George's, GRD; 2 Department of Medicine, St. George's University School of Medicine, St. George's, GRD

**Keywords:** community outreach, student organizations, equality, gender disparity, medical education, student role models, brain bee, grassroot neuroscience, near-peer engagement

## Abstract

Objective

This article explores the value of hosting a grassroots neuroscience workshop that facilitates near-peer engagement between year-one medical students and local Brain Bee finalists (high school students). Near-peer mentoring is a formal relationship in which more academically advanced students guide immediate junior students. We hypothesized that similar activities have teaching, learning, and psychosocial benefits for all and can be easily replicated.

Activity

The Grenada National Brain Bee Challenge was launched in 2009 as a competition for high school students. Annually, there are at least 100 high school students registering to participate in the national challenge. In 2018, a grassroots neuroscience symposium, a local initiative, was created to prepare high school students who participated in the preliminary rounds for the final local and International Brain Bee competition. Traditionally, it is hosted annually by faculty at St. George's University School of Medicine (SOM). However, in 2022, the symposium was hosted by medical students. The symposium is designed as an eight-hour tutorial one-day session. The students rotate between facilitators as small group teams during each teaching hour. There are icebreakers, content presentations, and neuroanatomy skills stations. The medical students demonstrate expertise in neuroscience content and other aspects of professional competence. The activity was also designed to offer students of diversified backgrounds the opportunity to affect their educational pathways through role modeling, mirroring, and mentorship. Was this change beneficial to both sets of students (medical and high school)?

Results and discussion

We aim to determine the value of the near-peer relationship between the local 2022 Brain Bee finalists (high school students) (n=28) and university (medical) students (n=11). Participants were surveyed about their experience. Data were de-identified and grouped according to common themes. A thematic analysis was conducted on the data retrieved from the literature review. Data suggest that both high school and university (medical) students report benefits after participating in near-peer engagement at a grassroots neuroscience symposium. In this teaching model, the medical students are the more experienced instructors and transfer their knowledge and skills about the field to the high school students. The medical students have an opportunity to consolidate their personal learning and give back to the Grenadian community. While informal teaching occurs often, this type of near-peer engagement with students from the community helps medical students develop both personal and professional skills such as confidence, knowledge, and respect. This grassroots initiative is easily replicated in a medical curriculum. The major benefits experienced by the high school student participants (of various socioeconomic backgrounds) were access to educational resources. The symposium requires active engagement, fosters a sense of belonging, and promotes interest in pursuing careers in health, research, academia, and Science, Technology, Engineering, and Mathematics (STEM).

Conclusion

Participating high school students of various genders and socioeconomic backgrounds gained equal access to educational resources and may select careers in health-related sciences. Participating medical students developed knowledge and teaching skills and engaged in a service-learning opportunity.

## Introduction

Various factors shape adolescent development, including parental and peer relationships, broader culture, and society. While adults typically serve as role models and mentors during this process, young adolescents tend to be more influenced by their peers than by adults. In either case, the role model can significantly impact their decisions, potentially leading to positive or negative changes [1]. Atif et al. suggest that the behaviors of role models and mentors influence significantly adolescents' development [1]. Although definitions of role models vary, a role model is commonly defined as the person youth look up to and aspire to be or resemble. Some studies have used the terms role model and mentor interchangeably. Traditionally, a mentor is a person who guides, supports, and encourages a mentee by interacting closely and regularly. Therefore, positive role modeling and mentorship are vibrant components in youth life to promote curiosity and engagement in Science, Technology, Engineering, and Mathematics (STEM). As the STEM fields are frequently underrepresented due to a lack of interest and exposure, offering access to various scientific fields during adolescence is crucial to enable informed decisions about educational and career paths.

In 1977, Albert Bandura proposed the Social Learning Theory, which suggests that behavior is learned through observation and the environment and that people of all ages use role models to shape their behavior [2]. Later, in 1986, this theory was expanded into the Social Cognitive Theory, highlighting that learning occurs within a social framework that involves continuous changes in individuals, interactions with their environment, and observing others' behaviors. Self-efficacy is a key concept in this theory that suggests that learning, career choices, and beliefs about success are socially learned. Several studies deliberated the association between positive role models and positive outcomes among adolescents. In qualitative research conducted with high school students in South Africa, Matshabane [3] (2016) suggested that guidance and direct role modeling facilitate career inspiration among young adults. This study demonstrated the impact of role models on the overall career and life decisions made by young people. Valero et al. [4] (2019) investigated the beneficial role of having a role model and social support on career development among native and migrant Swiss youth in a sample of 191 high school students and 500 youths enrolled in vocational education training (VET). Their findings supported the Social Cognitive Theory, in which social role models, social support, and cognitive self-efficacy enrich career development among youth. Lee et al. [5] (2021) investigated how role modeling affects the self-efficacy and flow state of adolescent athletes. Their findings indicated that role modeling through a mentor has an indirect positive effect on the flow state. They concluded that role modeling can improve the self-efficacy of adolescent athletes, and higher self-efficacy has a positive influence on the flow state.

The COVID-19 pandemic has underscored the importance of science and technology, creating a need for more professionals in STEM-related careers. A UN Women in Latin America and the Caribbean report suggests that there is underrepresentation of women in STEM-related fields when analyzing female tertiary graduates. As a result, initiatives have emerged to encourage young people, especially females and underrepresented minorities, to engage actively in science and pursue STEM careers.

Some examples of programs that target girls in the Caribbean are Technovation Girls (global technology program), Girl Power Codefest (code programming for girls), and Girls4Tech (STEM courses, e.g., fraud detective, data scientist, and software engineering). However, numerous studies have highlighted the gender gap in STEM fields, indicating that women are less likely than men to pursue science and technology-related disciplines, resulting in a shortage of STEM skills among female students [6]. In a research study, Hardin and Longhurst [7] (2016) found that female students had lower self-efficacy and interest in STEM than male students. Additionally, the study found that male students received slightly more support for pursuing STEM degrees than female students [7]. In addition, it was observed that men experienced slightly more support for pursuing a STEM degree than women. Guenaga et al. [8] (2022) studied the impact of female role models in leading a group mentoring program to enhance STEM vocations among young females. The study's results supported the impact of that program on increasing the number of female STEM references and improving their opinions of vocations related to science and technology. A systematic review of articles selected 28 studies investigating the effects of schools on students' STEM orientation [9]. The findings indicated a lack of structured career exploration support from advisors. Moreover, findings implicated that STEM teaching and learning through extracurricular activities, along with connecting with experts and practicing scientists, had more impact in implementing a clear understanding and strengthening students' orientation of STEM principles and career paths.

The Brain Bee program, established by Dr. Norbert Myslinski of the University of Maryland in 1998, has grown from a local high school neuroscience competition to an international program encouraging students' curiosity in neuroscience. The Brain Bee Challenge was introduced in Grenada in 2009 as the first grassroots neuroscience program in the Caribbean [10]. The aim of the program was to increase interest in neuroscience among high school students despite challenges that include a lack of neuroscience-trained teachers, a shortage of neuroscience-specific texts in school libraries, limited access to internet and computer facilities, and transportation-related issues, particularly in rural areas.

An innovative component of the initiative to promote equality among high school students in the local Brain Bee Challenge is the neuroscience symposium hosted by the local medical university faculty for participants. This one-day event includes content sessions in different neuroscience-related fields, hands-on engagement in the wet and dry laboratory, research expositions, and featured guest speakers. This initiative is essential as it addresses the challenges of underserved populations, lack of access to resources, and limited exposure to science. In Grenada, this initiative is considered “grassroots” and contributes to an increased interest in STEM and the promotion of equal opportunities for all students. Traditionally, the sessions were facilitated by medical doctors who were faculty of the medical school. However, in 2022, the local Brain Bee organizing committee partnered with the medical students of the Neuroscience Society of St. George's University. These medical students were volunteer first- and second-year students at St. George's University School of Medicine. The proposed change offered medical students the opportunity of service learning and teaching within the community. Data suggest that students rate the activity's interest higher when they learn to teach their peers rather than being tested and help promote interest in health careers and long-term valuable relationships [11,12]. Was this near-peer engagement beneficial to both sets of students (medical and high school)?

## Materials and methods

A total of five databases (PubMed, National Institutes of Health (NIH), JURN, ScienceDirect, and Google Scholar) were utilized to search for up-to-date, innovative approaches or programs to enhance adolescents' interest and engagement in the STEM fields. Key search words included "Brain Bee," "STEM programs," "learning environments," "public outreach and engagement," "education and career choice," "role modeling," "mentorship," "STEM disparities," "active learning," "public outreach," and "scientific practices." The studies selected for this paper are pertinent to its focus, which is to promote scientific interests and encourage engagement in STEM roles among young individuals. We extracted different study designs and articles demonstrating the importance of various innovative opportunities of broad science environments to enhance students' motivation and understanding of STEM careers.

An online survey was sent to all 2022 local Brain Bee finalists (n=28). This sample size was small and can be considered a pilot study. The survey was limited to the number of participants in the 2022 symposium since this symposium was the first one that offered near-peer engagement facilitated by medical students instead of faculty.

The survey was processed using an app called Google Forms (Google, Inc., Mountain View, CA, USA). The link was shared via a discussion thread on a school-based software, Canvas by Instructure (Instructure, Salt Lake City, UT, USA). A second survey was sent to all medical students who participated as facilitators (n=11) in the workshop. All participants gave their informed consent for inclusion before they participated in the study. The survey distributed to the high school finalists contained four questions and a small explanation as shown in Table 1.

**Table 1:** Survey questions for the local 2022 Grenada Brain Bee Challenge finalists (high school students) STEM: Science, Technology, Engineering, and Mathematics This table was created by the corresponding author (GWB).

Instructions: Please ask your parents for consent BEFORE answering any of the questions on this survey and submit your consent electronically. This survey is completely anonymous. Do not share your name or age on this form.						
BEFORE participating in the Brain Bee, did you consider pursuing a career in any Science, Technology, Engineering, and Mathematics (STEM) field?				Yes		No
AFTER participating in the Brain Bee, have you considered pursuing a career in any Science, Technology, Engineering, and Mathematics (STEM) field?				Yes		No
If you answered NO in question 1 and YES in question 2, please briefly tell us what made you change your mind.						
If you are comfortable, please indicate your gender preference. Please note you may decline to answer this question if you decide to do so.	Male	Female	Other		Did not specify	

 The survey for the facilitators contained two questions as shown in Table 2.

**Table 2:** Survey questions for local 2022 Brain Bee facilitators (medical students) This table was created by the corresponding author (GWB).

Survey questions				
1. On a scale from 1 to 5, (1 = not beneficial and 5 = very beneficial), how would you rate your Brain Bee experience toward your studies and near-peer interactions?				
1. Not beneficial	2. Somewhat not beneficial	3. Neutral	4. Somewhat beneficial	5. Very beneficial
2. In one sentence, please describe how you benefited from facilitating the event. You may use single-word adjectives to describe your experience.				

The survey questions were designed with three primary objectives: to gather feedback on the benefits of the neuroscience symposium, particularly regarding peer-to-peer interactions and active engagement, to establish a correlation between active exposure to mentors in STEM-related fields and increased choice to pursue STEM-related careers, and to ask high school students to indicate their gender, but they could decline to answer if they felt uncomfortable sharing this information. The purpose was to examine any differences in gender concerning influence and decision-making on career choices. The Results section displays the data collected from the surveys.

## Results

Thematic analysis was conducted on the data retrieved from the literature review. The main themes that emerged from the literature review were student-facilitator benefits and high school student benefits. Table 3 shows the impact of mentorship and role modeling on promoting the youth's interest in complex topics [12]. Furthermore, the current data shows the importance of active engagement in STEM-related fields to promote the inherent interest of the exposed subject and generate a positive impact [13-17].

**Table 3:** Study designs and thematic analysis results from the literature review STEM: Science, Technology, Engineering, and Mathematics This table was created by the co-author (ENG).

Author(s)	Source	Design	Number	Major findings
Frey et al. [13]	Medical Science Educator	Qualitative research method assessing the impact of early introduction to neurosciences through the Brain Bee competition	34	Findings illustrated increased high school student interest in neuroscience. In addition, it revealed the significance of role models and mentorship for student learning.
Dowie et al. [14]	The Neuroscientist	Qualitative research aimed to determine the influence of a neuroscience outreach program on the highest achievers' choices in education, career expectations, and their perspectives on science	7	The study acknowledged the importance of diverse opportunities for individual development in which the Brain Bee challenge increased students' exposure to broad science environments.
Chittum et al. [15]	International Journal of STEM Education	The research used longitudinal quantitative data assessing the impact of an afterschool STEM program on students' interest and engagement from two samples of students who enrolled in the program and those who did not	121	The study found that for students who were enrolled in the program, their experience had a positive impact on their perceptions of science as a field and increased their motivational beliefs about science.
Drymiotou et al. [16]	International Journal of Science Education	A case study approach of a group of secondary school students exploring the impact of career-based scenarios in enhancing students' interest and understanding of STEM careers	16	The findings demonstrated the importance of opportunities for active engagement in scientific practices with experts in increasing students' interest in science and understanding of STEM careers.
Hiğde et al. [17]	Thinking Skills and Creativity	Mixed research design aiming to determine the effects of STEM activities on secondary school students' interests and views on STEM education	44	The findings revealed that STEM activities enhanced students' STEM career interests and motivation, and improved students' science process skills. Additionally, it showed that STEM activities developed positive implications on skill sets such as creativity, collaboration, critical thinking, and problem-solving.

**Figure 1:**
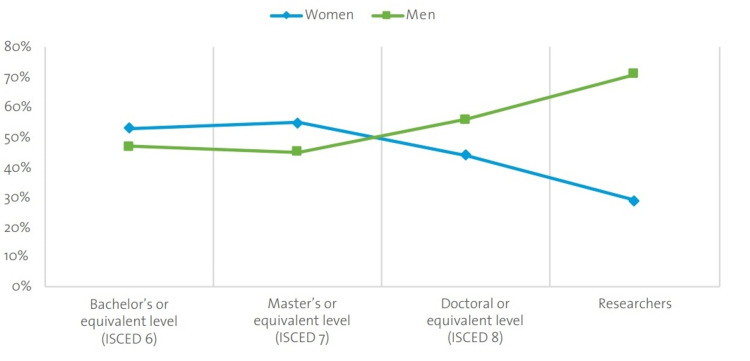
Proportion of female and male graduates in tertiary education by program level and those employed as researchers (global estimate, 2017 or latest year available) (based on the UIS, US data (July 2019)) UNESCO: United Nations Educational, Scientific and Cultural Organization, UIS: UNESCO Institute for Statistics, ISCED: International Standard Classification of Education Image source: https://lac.unwomen.org/sites/default/files/Field%20Office%20Americas/Documentos/Publicaciones/2020/09/Women%20in%20STEM%20UN%20Women%20Unesco%20EN32921.pdf

Additionally, while more women are enrolling in university, the attrition rate at higher levels of education is greater in women than men. Figure 1 shows that Latin America and the Caribbean is one of the two regions that achieved parity in the percentage of female and male researchers. The grassroots initiative fits within the culture of the Caribbean to facilitate gender equity in STEM-related fields.

**Figure 2:**
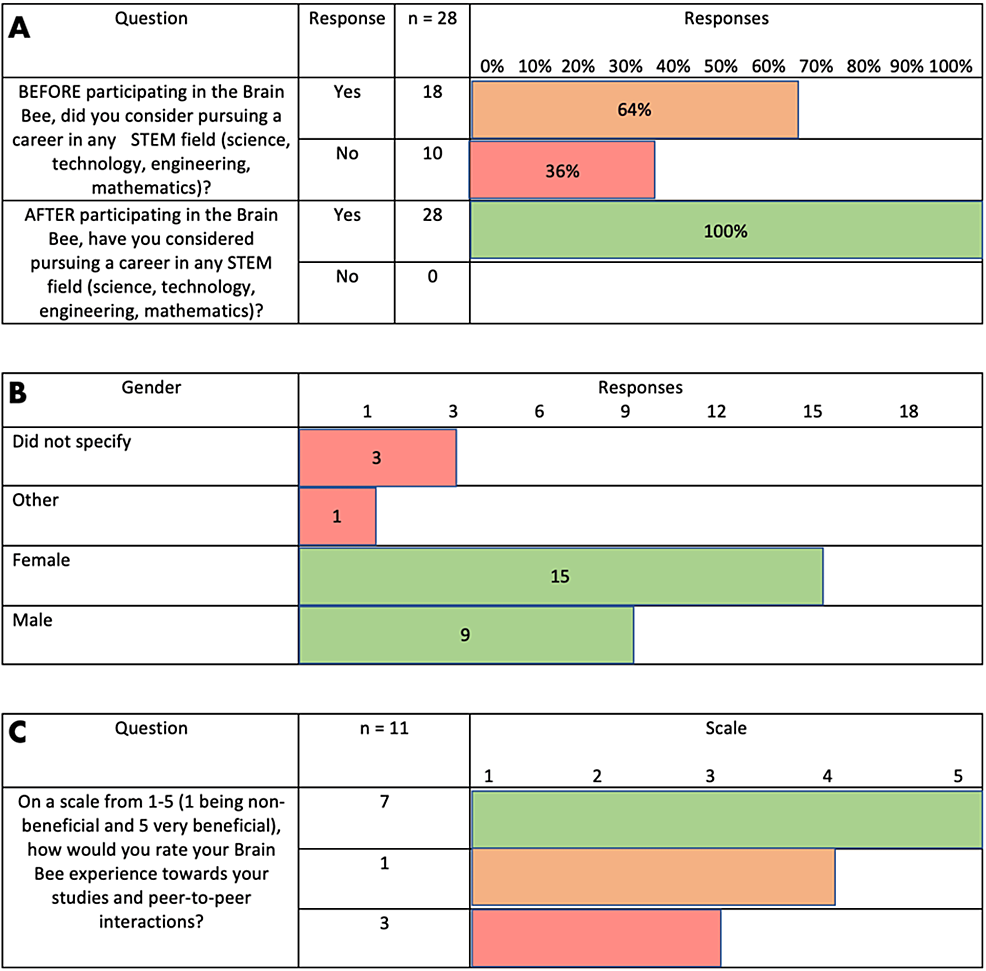
All survey results: (A) interest in STEM-related careers before and after participating in the Brain Bee, (B) gender distribution of Brain Bee participants, and (C) benefit rating indicated by all the facilitators STEM: Science, Technology, Engineering, and Mathematics This image was created by the co-author (ENG).

The data in Figure 2A corroborates the importance of near-peer engagement and active exposure to STEM-related fields. Before participating in the Brain Bee, only 64.3% of the participants had decided to pursue a career in STEM. After the Brain Bee concluded, 100% of the participants decided to pursue a field of study in a STEM-related career. This is a 35.7% increase just by actively engaging in the field.

The data in Figure 2B highlights the importance of the Brain Bee and other events in addressing gender disparities. Of the participants in this event, 51.7% are women. This data strongly suggests the importance of giving equal access to all genders in STEM fields, particularly at a young age. Both participants and facilitators indicated that shared "common goals," "interacting with like-minded people," "seeing their mentors succeed,” “practicing teaching skills,” and “mastering content” were some of the reasons that largely contributed to their feeling of satisfaction with the event. This shared feeling of "community" enhances students' learning abilities by providing support and guidance through complex topics.

The data in Figure 2C shows how valuable this event is to the student body, which further exemplifies how meaningful near-peer interactions are at all ages and during various stages of education. Overall, the data collected strongly validates the importance of organizing events where underrepresented social classes, genders, and different group ages interact with well-established scientists and students pursuing STEM careers. These interactions positively impact the development of all those involved.

## Discussion

Benefit to the high school students

Continuing advancements in science and technology, coupled with the growing need for a workforce well-versed in STEM, have made it crucial for students to engage in STEM-based learning practices on a global scale [17,18]. Several studies have supported that engagement in STEM programs and activities, where students have opportunities for hands-on learning and real-life applications, alongside guidance from experts and mentors, has resulted in increased interest in STEM fields [15,16,19,20]. The Brain Bee programs held globally have generated a substantial increase in interest in STEM fields due to ongoing exposure to various neuroscientific topics. While some studies demonstrate the Brain Bee's influence in promoting the pursuit of careers in neuro-specific fields, our data indicate that early exposure to neuroscience and events such as the Brain Bee can stimulate an interest in all STEM-related fields, not just neuroscience. The success of the Brain Bee program can be attributed to the near-peer interactions between young participants. Active exposure increases the level of interest that students have in any topic [15]. Being exposed to significant STEM-related events where students can interact with like-minded individuals increases their likelihood of pursuing a STEM-related career. This shared feeling of community and sense of belongingness has been established and proven to show a positive impact on the experience felt by the participants [21].

Furthermore, this initiative's impact on young women is also considerable, given that most of the survey responses were from women. This event directly tackles the gender disparity that exists in the STEM field. This study further amplifies the benefit of major psychological roles in shaping the youth.

Benefit to the medical school students

According to the liaison committee on medical education in the United States, medical students must develop a culture of lifelong teaching and development of professional skills. This initiative offered medical students a unique opportunity to put their basic science knowledge and skills into practice. When medical students assume a teaching role, they can develop confidence and feelings of self-efficiency [20]. As indicated previously, medical students acting in a role as peer experts can encourage increased personal study and mastery of the content as they prepare to "teach" high school students. The medical students created PowerPoint teaching resources that can be reused by their peers in upcoming sessions. In contrast to the large lecture teaching, the sessions were in the form of small groups. This mode increased engagement and interaction between both the student and the facilitator. Exposure to the enthusiasm of high school students may strengthen their own personal desires to pursue medicine.

Limitations

There were several limitations identified. The sample size of students (both participants and facilitators) was small. There was nothing more that could have been done to increase the sample size since the symposium was limited to only the local Brain Bee finalists. Maybe, in the future, the symposium could be open to all of the participants who met the preliminary qualifying criteria for the competition. Therefore, their perspective may not be generalizable to the larger population. There was no standardized training for the student facilitators on how to teach. Students relied on the resource text and their personal notes for handout creation. It is recommended that a training session for the peers be held prior to the symposium. The activity relied on the fact that the medical student volunteers were high-performing students who were active members of the Neuroscience club. They were familiar with the content in much greater detail. It can be argued that students who have not participated in student organizations would be excluded from the opportunity. This can be mitigated if the opportunity is well advertised among the student population. The study sample collected responses after only one iteration of the activity. This study should be continued annually at least for five more iterations. One cannot say if their interest or perspectives would change over time based on other external factors such as financial or social issues. Future research could include a longitudinal study to track if the interest did result in the pursuit of a job within the STEM-related field. It would also be interesting to compare the perspectives of students who participated in the faculty-run symposium with the perspectives of students in the student-run symposium.

## Conclusions

With the increasing need for medical students to develop personal and professional skills, medical school curricula must include opportunities for near-peer engagement within the community as well as in the classroom. This grassroots initiative is easily replicated and may provide numerous benefits to high school students, especially if it creates near-peer engagement with medical students. The major benefits experienced by the high school student participants were promoting STEM interest among youth to tackle educational disparities related to variable socioeconomic status. The event's open format encourages near-peer interactions and active engagement, fostering a sense of belonging and promoting interest in pursuing careers in healthcare, research, academia, and other STEM fields. Mentorship and role modeling are critical in developing young scientists as part of a scientific community. Establishing a partnership between the high school students identified by the local Brain Bee organizing committee and the medical students of the Neuroscience Society within the University can become a sustainable initiative to foster near-peer engagement with tremendous benefit.
